# High-quality draft genome sequences of five anaerobic oral bacteria and description of *Peptoanaerobacter stomatis* gen. nov., sp. nov., a new member of the family *Peptostreptococcaceae*

**DOI:** 10.1186/s40793-015-0027-8

**Published:** 2015-07-18

**Authors:** Maria V. Sizova, Amanda Chilaka, Ashlee M. Earl, Sebastian N. Doerfert, Paul A. Muller, Manolito Torralba, Jamison M. McCorrison, A. Scott Durkin, Karen E. Nelson, Slava S. Epstein

**Affiliations:** Northeastern University, 360 Huntington Avenue, Boston, MA USA; Genome Sequencing and Analysis Program, Broad Institute of MIT and Harvard, 7 Cambridge Center, Cambridge, MA USA; J. Craig Venter Institute, 9704 Medical Center Dr., Rockville, MD USA

**Keywords:** *Peptostreptococcaceae*, *Peptoanaerobacter stomatis*, Uncultivable bacteria, Anaerobic oral bacteria, Human oral taxa

## Abstract

Here we report a summary classification and the features of five anaerobic oral bacteria from the family *Peptostreptococcaceae*. Bacterial strains were isolated from human subgingival plaque. Strains ACC19a, CM2, CM5, and OBRC8 represent the first known cultivable members of “yet uncultured” human oral taxon 081; strain AS15 belongs to “cultivable” human oral taxon 377. Based on 16S rRNA gene sequence comparisons, strains ACC19a, CM2, CM5, and OBRC8 are distantly related to *Eubacteriumyurii* subs. *yurii* and *Filifactor alocis*, with 93.2 – 94.4 % and 85.5 % of sequence identity, respectively. The genomes of strains ACC19a, CM2, CM5, OBRC8 and AS15 are 2,541,543; 2,312,592; 2,594,242; 2,553,276; and 2,654,638 bp long. The genomes are comprised of 2277, 1973, 2325, 2277, and 2308 protein-coding genes and 54, 57, 54, 36, and 28 RNA genes, respectively. Based on the distinct characteristics presented here, we suggest that strains ACC19a, CM2, CM5, and OBRC8 represent a novel genus and species within the family *Peptostreptococcaceae*, for which we propose the name *Peptoanaerobacter stomatis* gen. nov., sp. nov. The type strain is strain ACC19a^T^ (=HM-483^T^; =DSM 28705^T^; =ATCC BAA-2665^T^).

**Electronic supplementary material:**

The online version of this article (doi:10.1186/s40793-015-0027-8) contains supplementary material, which is available to authorized users.

## Introduction

The oral cavity is a major gateway to the human body [1] and one of the principle sites of interest to the Human Microbiome Project, which aims to characterize this microbiome and understand its role in health and disease.

The 16S rRNA surveys and metagenomic analyses indicate that the typical oral community is comprised of over 700 bacterial species [2–4], approximately half of which have been isolated in culture and formally named. The rest remain uncultivated or unclassified [1, 5]. Anaerobic species are of particular importance as they constitute approximately one half of the human oral microbiome [6–8] and likely play an important role in the function of the oral microbial community.

The Human Oral Microbiome Database, provides comprehensive information on currently known prokaryote species and presents a provisional “oral taxa” naming scheme for the presently unnamed cultivable and uncultivable species. HOMD also provides links to genome sequencing projects of oral bacteria [9]. There are annotated genomes for 381 oral taxa currently available at HOMD.

Five anaerobic strains ACC19a, CM2, CM5, OBRC8, and AS15 from the family *Peptostreptococcaceae* were isolated earlier from the subgingival plaque obtained from two young African American and two young Caucasian females. Cultivation techniques were described before [10].

Family *Peptostreptococcaceae* currently is represented by five validly-named genera, *Anaerosphaera**,**Filifactor**,**Peptostreptococcus**,**Sporacetigenium**,* and *Tepidibacter* [11, 12], and several unclassified species. At this time, genome sequences of oral bacteria from the family *Peptostreptococcaceae* are available for three strains of *Peptostreptococcus anaerobius*, one strain of *P. stomatis*, one strain of *Filifactor alocis**,* and one strain of unclassified *Eubacterium**yurii* subsp. *margaretiae*.

According to HOMD, the genera *Peptostreptococcus* and *Filifactor* are represented by three oral taxa, while the other eleven *Peptostreptococcaceae* oral taxa remain formally unclassified. To date, only two unclassified oral taxa are represented by cultivable isolates, whereas nine stay “yet uncultured” and are known only by their molecular signatures. Strains ACC19a, CM2, CM5, and OBRC8 described here represent the first known cultivable members of “yet uncultured” human oral taxon 081; strain AS15 is classified as a member of “cultivable” oral taxon 377.

Here we report a summary classification and the features of strains ACC19a, CM2, CM5, OBRC8, and AS15 together with their genome sequence and annotation. Strains have been deposited in BEI Resources, ATCC and DSMZ under deposition numbers HM-483, DSM 28705, ATCC BAA-2665 (for ACC19a), HM-484, DSM 28703, ATCC BAA-2664 (for CM2), HM-485, DSM 28704 (for CM5), HM-765, DSM 28706 (for OBRC8), and HM-766, DSM 28702, ATCC BAA-2661 (for AS15) respectively.

## Organism information

### Classification and features

**Fig. 1:**
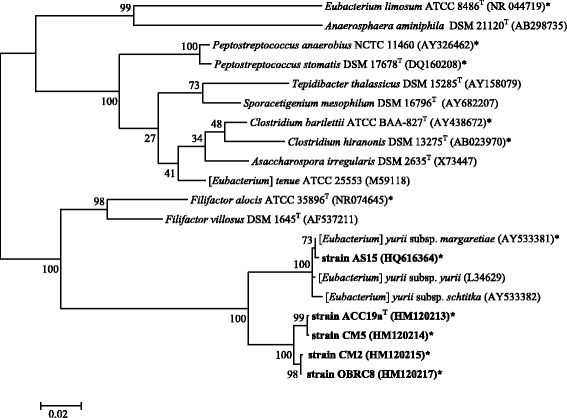
Maximum-Likelihood phylogenetic tree based on 16S rRNA gene sequence comparisons of strains ACC19a, CM2, CM5, OBRC8, and AS15 (shown in bold) together with other representatives of the *Peptostreptococcaceae* family and other related human bacteria. The tree was derived based on Tamura-Nei model using MEGA 5 [39]. Bootstrap values > 50 % calculated for 1000 subsets are shown at branch-points. Bar 0.02 substitutions per position. Strains whose genomes have been sequenced are marked with an asterisk

Phylogenetic analysis based on 16S rRNA gene sequence comparisons showed that strains ACC19a, CM2, CM5, and OBRC8 were only distantly related to *Eubacterium**yurii* subs. *yurii*, *E.yurii* subs. *schtitka,E.yurii* subsp. *margaretiae* and *Filifactor alocis**,* and formed a separate branch within the *Peptostreptococcaceae*, while strain AS15 was closely related *E.yurii* subsp. *margaretiae* (Fig. 1). The validly published species of *E.yurii* subs. *yurii*, *E.yurii* subs. *schtitka* and [*E.*] *yurii* subs. *margaretiae* have historically been misclassified and were included within the genus *Eubacterium* [13, 14], but according to 16S rRNA gene sequence phylogeny, [*E.*] *yurii* falls into the *Peptostreptococcaceae* [15].

**Fig. 2:**
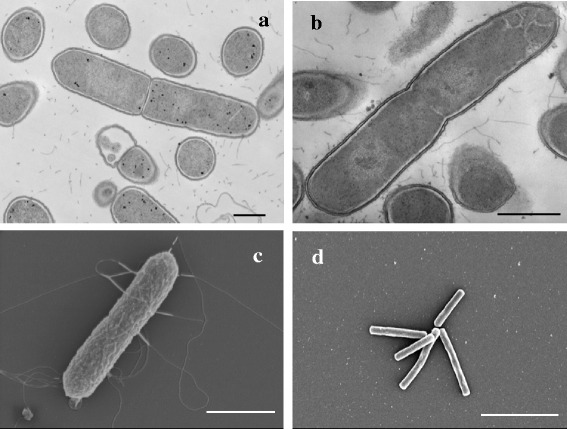
Transmission and scanning electron micrographs of anaerobic oral bacteria from the family *Peptostreptococcaceae.* General morphology and Gram-positive cell wall structure of strains CM5 (**a**) and ACC19a (**b**), peritrichous flagella of strain CM2 (**c**), rosettes or brush-like structures formed by strain AS15 (**d**). Bars, 500 nm (**a, b**), 1 μm (c) and 5 μm (**d**)

Cells of strains ACC19a, CM2, CM5, and OBRC8 are non-spore-forming, highly motile, peritrichous rods with round ends; cells often form chains. Cells of strain AS15 are motile, monotrichous, straight rods with square ends that often form rosettes or brush-like aggregates (Table 1, Fig. 2). On liquid TY medium, cells of strains ACC19a, CM2, CM5, and OBRC8 range from 1.0 to 3.4 μm in length and from 0.4 to 0.8 μm in width; cells of strain AS15 are 1.5 – 4.7 μm long and 0.4 - 0.5 μm wide (Table 1, Fig. 2). Cells are Gram-positive, structurally and by staining (Table 1, Fig. 2). After 48-72 h incubation on TY blood agar plates at 37 °C, strains ACC19a, CM2, CM5, and OBRC8 formed pin-point, beige, circular, convex, non-hemolytic colonies, approximately 0.5 mm in diameter. Colonies of strain AS15 are circular, umbonate, alpha-hemolytic, yellow-greenish in pigment, 1 mm in diameter after 48-72 h, and 2-3 mm in diameter after 168 h.

**Table 1:** Classification and general features of the five oral isolates according to the MIGS recommendation [34]

MIGS ID	Property	Term					Evidence code^a^
		strain ACC19a	strain CM2	strain CM5	strain OBRC8	strain AS15	
	Classification	Domain *Bacteria*	Domain *Bacteria*	Domain *Bacteria*	Domain *Bacteria*	Domain *Bacteria*	TAS [35]
		Phylum *Firmicutes*	Phylum *Firmicutes*	Phylum *Firmicutes*	Phylum *Firmicutes*	Phylum *Firmicutes*	TAS [36]
		Class *Clostridia*	Class *Clostridia*	Class *Clostridia*	Class *Clostridia*	Class *Clostridia*	TAS [36]
		Order *Clostridiales*	Order *Clostridiales*	Order *Clostridiales*	Order *Clostridiales*	Order *Clostridiales*	TAS [36]
		Family *Peptostreptococcaceae*	Family *Peptostreptococcaceae*	Family *Peptostreptococcaceae*	Family *Peptostreptococcaceae*	Family *Peptostreptococcaceae*	IDA
		Genus *Peptoanaerobacter*	Genus *Peptoanaerobacter*	Genus *Peptoanaerobacter*	Genus *Peptoanaerobacter*	Genus *Eubacterium*	IDA
		Species *Peptoanaerobacter stomatis*	Species *Peptoanaerobacter stomatis*	Species *Peptoanaerobacter stomatis*	Species *Peptoanaerobacter stomatis*	Species *Eubacterium* *yurii* subspecies *margareti*ae	IDA
		Type strain HM-483; DSM 28705; ATCC BAA-2665					TAS [10]
	Gram stain	Positive	Positive	Positive	Positive	Positive	IDA
	Cell shape	Rods with round ends	Rods with round ends	Rods with round ends	Rods with round ends	Rods with square ends, forms rosettes	IDA
	Cell size, μm	0.4-0.8 × 1.2-2.5	0.5-0.7 × 1.0-2.3	0.5-0.7 × 1.3-2.8	0.6-0.8 × 1.4-3.5	0.4-0.5 × 1.5-4.7	IDA
	Motility/Flagella	+/peritrichous	+/peritrichous	+/ peritrichous	+/ peritrichous	+/single subpolar	IDA
	Sporulation	Does not form spores	Does not form spores	Does not form spores	Does not form spores	Does not form spores	IDA
	Temperature range	30 – 42 ^o^C	30 – 42 ^o^C	30 – 42 ^o^C	30 – 42 ^o^C	30 – 42 ^o^C	IDA
	Optimum temperature	37 °C	37 °C	37 °C	37 °C	37 °C	IDA
	pH range; Optimum	6.5-7.5; 7	6.5-7.5; 7	6.5-7.5; 7	6.5-7.5; 7	6.5-7.5; 7	IDA
	Carbon source	Yeast extract	Yeast extract, Glucose, Sucrose, Maltose	Yeast extract	Yeast extract, Glucose, Sucrose, Maltose	Yeast extract, Glucose, Sucrose, Maltose	IDA
MIGS-6	Habitat	Human oral cavity					TAS [10]
MIGS-6.3	Salinity	Normal					IDA
MIGS-22	Oxygen requirement	Strictly anaerobic					TAS [10]
MIGS-15	Biotic relationship	Free living					TAS [10]
MIGS-14	Pathogenicity	Non pathogen					TAS [10]
MIGS-4	Geographic location	Boston, Massachusetts, USA					TAS [10]
MIGS-5	Sample collection	March 1, 2010					TAS [10]
MIGS-4.1	Latitude	42.34					NAS
MIGS-4.2	Longitude	−71.09					NAS
MIGS-4.4	Altitude	5.8 m above see level					NAS

^a^Evidence codes - IDA: Inferred from Direct Assay; TAS: Traceable Author Statement (*i.e.*, a direct report exists in the literature); NAS: Non-traceable Author Statement (*i.e.*, not directly observed for the living, isolated sample, but based on a generally accepted property for the species, or anecdotal evidence). These evidence codes are from Gene Ontology project [37, 38]

Isolated strains grew only under strict anaerobic conditions. Growth occurred from 30 to 42 °C, with optimum growth at 37 °C. All isolates were susceptible to discs containing 1 mg kanamycin, 2 units penicillin, 60 μg erythromycin, 30 μg chloramphenicol, 30 μg tetracycline and bile. Catalase, oxidase and urease activities were negative; nitrate reduction was not detected, gelatin was not liquefied, and aesculin was not hydrolyzed. Strains ACC19a, CM2, CM5, and OBRC8 did not produce indole, while strain AS15 did produce indole (Table 1). All strains were able to grow on 2.0 – 10 g l^−1^ of yeast extract, but not on casamino acids. No visible biomass was formed in medium with 0.5 – 2.0 g l^−1^ of yeast extract only. All five strains produced acid on API 20A media containing glucose, maltose and sucrose, but not lactose, arabinose, cellobiose, mannose, melezitose, raffinose, rhamnose, trehalose, xylose, glycerol, mannitol, salicin and sorbitol. All produced gas on TY liquid medium. In liquid medium, supplemented with 5.0 g l^−1^ of yeast extract, strains CM2, OBRC8 and AS15 fermented D-glucose, D-sucrose and D-maltose; strains ACC19a, CM2, CM5 and OBRC8 poorly fermented L-glutamine; strain CM2 fermented L-serine; strains ACC19a, CM5, and AS15 weakly fermented L-alanine; strains CM2, CM5, and AS15 poorly fermented L-valine. The major metabolic end products of strains ACC19a, CM2, and CM5 on TY medium were acetate and propionate (Table 1).

Cell biomass that was grown in TY liquid medium for 48 h was used for the whole-cell fatty acids analysis. Fatty acids were methylated, extracted, and analyzed by GC using the Sherlock Microbial Identification System at Microbial ID, Inc. Fatty acid methyl esters profile showed that strain ACC19a contained C12:0 (5.6 %), C14:0 (46.6 %), C16:0 (7.8 %), C16:1ω7c (9.4 %), and C16:1ω7c DMA (5.2 %) as major fatty acids; strain CM2 contained C 12:0 (5.2 %), C14:0 (47.1 %), C16:0 (5.7 %), C16:1ω7c (6.9 %), and C16:1ω7c DMA (7.2 %); and strain CM5 contained C14:0 (40.6 %), C16:0 (7.4 %), C16:1ω7c (11.5 %), and C16:1ω7c DMA (6.8 %) (Table 1). Genomic DNA G + C content of strains ACC19a, CM5, CM2 and OBRC8 was between 30.0 – 30.7 %, and of strain AS15 was 32.2 % (Table 2).

## Genome sequencing information

### Genome project history

The genomes were selected for sequencing in 2010-11 by the HMP. For strains ACC19a, CM2, and CM5, sequencing, finishing, and annotation were performed by the Broad Institute of Harvard and MIT. For strains OBRC8 and AS15, sequencing, finishing, and annotation were performed by the J. Craig Venter Institute (JCVI). The genomes were deposited in the Genome On-Line Database [16]; the complete genome sequences were deposited in GenBank and are available in the RefSeq database [17–19]. Project information and association with MIGS version 2.0 is presented in Table 3. The genome finishing quality for all strains was High-Quality Draft.

**Table 2:** Genomes statistics

Attribute	strain ACC19a		strain CM2		strain CM5		strain OBRC8		strain AS15	
	Value	%^a^	Value	%^a^	Value	%^a^	Value	%^a^	Value	%^a^
Genome size (bp)	254, 1543	100	231, 259, 2	100	259, 424, 2	100	255, 327, 6	100	265, 463, 8	100
DNA coding region (bp)	215, 2064	85	196, 164, 0	85	219, 838, 6	85	217, 178, 3	85	220, 441, 4	83
DNA G + C (bp)	771, 857	30	695, 842	30	790, 067	30	783, 396	31	855, 775	32
DNA scaffolds	59	100	19	100	106	100	40	100	52	100
Total genes	2, 331	100	2, 030	100	2, 379	100	2, 313	100	2, 336	100
Protein coding genes	2, 277	98	1, 973	97	2, 325	98	2, 277	98	2, 308	99
RNA genes	54	2	57	3	54	2	36	2	28	1
Pseudo genes	0	0	0	0	0	0	0	0	0	0
Genes in internal clusters	21	1	4	0	18	1	4	0	10	0
Genes with function prediction	1, 811	78	1, 618	80	1, 873	79	1, 868	81	1, 915	82
Genes assigned to COGs	1, 404	60	1, 362	67	1, 448	61	1, 422	61	1, 472	63
Genes with Pfam domains	1, 856	80	1, 636	81	1, 917	81	1, 822	79	1, 851	79
Genes with signal peptides	129	6	120	6	130	6	131	6	174	7
Genes with transmembrane helices	531	23	455	22	505	21	514	22	616	26
CRISPR repeats	0	0	0	0	0	0	0	0	0	0

^a^% - Percent of total. The total is based on either the size of the genome in base pairs or the protein coding genes in the annotated genome

### Growth conditions and genomic DNA preparation

Strains ACC19a, CM2, CM5, OBRC8, and AS15 were cultivated on liquid TY anaerobic medium as previously described [10].

Genomic DNA was extracted from microbial biomass with the PowerMicrobial® Maxi DNA Isolation Kit (MO BIO Laboratories, Inc.) using phenol: chloroform in combination with bead beating cell lysis.

### Genome sequencing and assembly

Strains ACC19a, CM2, and CM5 were sequenced using two 454 pyrosequence libraries on the 454 platform: one standard 0.6 kb fragment library and one 2.5 kb jump library [20]. Library construction and sequencing process details are available at www.broadinstitute.org and 454 technologies. For strain CM2, additional sequence data was generated using two Illumina libraries on the Illumina HiSeq 2000 platform: one standard 180 bp fragment library and one 3-5 kb jump library. Library construction and sequencing process details are available at www.broadinstitute.org. Strains ACC19a and CM5 454 data set was assembled using Newbler Assembler version 2.3 PostRelease-11/19/2009 and CM2 data sets were assembled using ALL-PATHS version R39099 (Table 3).

All three assemblies are considered High-Quality Draft and consist of: 59 contigs with a total size of 2,541,543 bases for strain ACC19a; 106 contigs with a total size of 2,594,242 bases for strain CM5; and 19 contigs with a total size of 2,312,592 bases for strain CM2. The error rates of the draft genome sequences for strains ACC19a and CM5 are estimated to be less than one in 10,000 (accuracy of ~ Q40) and less than 1 in 1,000,000 (accuracy of ~ Q60) for strain CM2. Average sequence coverage for strains ACC19a and CM5 is 40× and 39×, respectively, and 282× for strain CM2 (Tables 3, 4 and 2, Additional file 1: Table S1).

**Table 3:** Project information

MIGS ID	Property	Term				
		strain ACC19a	strain CM2	strain CM5	strain OBRC8	strain AS15
MIGS-31	Finishing quality	High-Quality Draft Genome Sequence				
MIGS-28	Libraries used	Two 454 pyrosequencing libraries, one standard 0.6 kb fragment library and one 2.5 kb jump library	Two Illumina libraries, one standard 180 bp fragment library and one 3-5 kb jump library	Two 454 pyrosequencing libraries, one standard 0.6 kb fragment library and one 2.5 kb jump library	Standard Illumina paired-end library	Standard Illumina paired-end library
MIGS-29	Sequencing platforms	454 FLX Titanium	Illumina HiSeq 2000	454 FLX Titanium	Illumina HiSeq 2000	Illumina HiSeq 2000
MIGS-31.2	Fold coverage	40×	282×	39×	32×	31×
MIGS-30	Assemblers	Newbler v.2.3	ALLPATHS v. R39099	Newbler v.2.3	Celera Assembler v.6.1	Celera Assembler v.6.1
MIGS-32	Gene calling method	PRODIGAL	PRODIGAL	PRODIGAL	GLIMMER	GLIMMER
	Locus Tag	HMPREF9629	HMPREF9630	HMPREF9628	HMPREF1143	HMPREF1142
	GenBank ID	AFZE00000000	AFZF00000000	AFZG00000000	ALNK00000000	ALJM00000000
	GenBank Date of Release	Dec 19, 2011	Dec 14, 2011	Dec 19, 2011	Aug 27, 2012	Aug 13, 2012
	GOLD ID	Gi06852	Gi06853	Gi06851	Gi09663	Gi09662
	BIOPROJECT	49887	49889	49891	78565	78563
MIGS 13	Source Material Identifier	HM-483; DSM 28705; ATCC BAA-2665	HM-484; DSM 28703; ATCC BAA-2664	HM-485; DSM 28704	HM-765; DSM 28706	HM-766; DSM 28702; ATCC BAA-2661
	Project relevance	Human Microbiome Project

**Table 4:** Summary of the genomes: one chromosome each and no plasmids

Strain	Label	Size	Topology	INSDC identifier	RefSeq ID
ACC19a	Chromosome	2.54	circular	AFZE00000000.1	NZ_AFZE00000000.1
CM2	Chromosome	2.31	circular	AFZF00000000.2	NZ_AFZF00000000.2
CM5	Chromosome	2.59	circular	AFZG00000000.1	NZ_AFZG00000000.1
OBRC8	Chromosome	2.55	circular	ALNK00000000.1	NZ_ALNK00000000.1
AS15	Chromosome	2.65	circular	ALJM00000000.1	NZ_ALJM00000000.1

Strains OBRC8 and AS15 were sequenced using Illumina paired-end sequencing technology on the Illumina HiSeq 2000 platform: one standard Illumina paired-end library. Library construction and sequencing process details are available at www.jcvi.org. Strains OBRC8 and AS15 Illumina data sets were assembled using Celera Assembler version 6.1.

Both assemblies are considered High-Quality Draft and consist of: 40 contigs with a total size of 2,553,276 bases for strain OBRC8 and 52 contigs with a total size of 2,654,638 bases for strain AS15. The error rates of the draft genome sequences for strains OBRC8 and AS15 are estimated to be less than 0.03 or 3 %. Average sequence coverage for strains OBRC8 and AS15 is 32× and 31×, respectively (Tables 3, 4 and 2, Additional file 1: Table S1).

Assessment of coverage, GC content, contig BLAST and 16S rRNA gene classification was consistent with the expected organism for all five genomes.

### Genome annotation

Strains ACC19a, CM2, and CM5 were annotated using PRODIGAL [21] with no additional manual curation performed. For strains OBRC8 and AS15, genes were identified using GLIMMER, also with no additional manual curation. Table 2 summarizes statistics for each genome, including gene count, according to the original annotations and the Integrated Microbial Genomes (IMG) and Metagenomes website as of May 15, 2014 [22]. Additional annotations using RAST were performed for comparison [23].

## Genome properties

Strains ACC19a, CM2, CM5, OBRC8, and AS15 genomes include one circular chromosome of 2,541,543; 2,312,592; 2,594,242; 2,553,276; and 2,654,638 bp, respectively, with DNA G + C content of 30.0 – 32.2 % (Table 4 and 2). The genomes comprise 2277, 1973, 2325, 2277, and 2308 protein-coding genes, respectively, and 54, 57, 54, 36, and 28 RNA genes, respectively. The coding regions accounted for 83.0 – 85.1 % of the genomes for all isolates (Table 2). The total number of genes ranged between 2030 and 2379 and the percent of genes assigned to clusters of orthologous groups (COGs) ranged from 60.2 % - 67.1 % (Table 2). The isolate with the smallest genome size, strain CM2, had the least number of predicted total genes and protein-coding genes, but the highest percentage of genes assigned to COGs. The percentage of genes with signal peptides for strains ACC19a, CM2, CM5, and OBRC8 ranged between 5.5 – 5.9 %; for strain AS15 the percentage was 7.45 %. The percentage of genes with transmembrane helices for strains ACC19a, CM2, CM5, and OBRC8 ranged between 21.2 – 22.8 %; for strain AS15 the percentage was 26.4 % (Table 2).

COG values for the annotation data directly from the sequencing centers were found on the IMG website, as of May 15, 2014 (Table 5). The percentages in Table 5 are the number of COG proteins out of the total number of annotated genes. For all strains, 32.9 % - 39.8 % of the proteins were not predicted to be part of a COG category; strain ACC19a had the highest percentage of proteins unassigned (Table 5). Strain CM2 had the highest sequence coverage, at 282×, and the lowest percentage of unassigned proteins, at 32.9 % (Table 3 and 5).

**Table 5:** Number of genes associated with general COG functional categories obtained from BROAD or JCVI pipelines

Code	Description	strain ACC19a		strain CM2		strain CM5		strain OBRC8		strain AS15	
		Value	%^a^	Value	%^a^	Value	%^a^	Value	%^a^	Value	%^a^
J	Translation, ribosomal structure and biogenesis	136	5.8	132	6.5	136	5.7	136	5.9	142	6.1
A	RNA processing and modification	0	0	0	0	0	0	0	0	0	0
K	Transcription	105	4.5	97	4.8	105	4.4	106	4.6	105	4.5
L	Replication, recombination and repair	111	4.8	96	4.7	145	6.1	113	4.9	104	4.5
B	Chromatin structure and dynamics	0	0	0	0	0	0	0	0	1	0
D	Cell cycle control, cell division, chromosome partitioning	23	1	22	1.1	23	1	22	1	21	0.9
V	Defense mechanisms	46	2	34	1.7	42	1.8	41	1.8	55	2.4
T	Signal transduction mechanisms	77	3.3	75	3.7	78	3.3	80	3.5	77	3.3
M	Cell wall/membrane/envelope biogenesis	70	3	67	3.3	69	2.9	73	3.2	71	3
N	Cell motility	56	2.4	50	2.5	52	2.2	48	2.1	55	2.4
U	Intracellular trafficking, secretion, and vesicular transport	40	1.7	33	1.6	35	1.5	37	1.6	41	1.8
O	Posttranslational modification, protein turnover, chaperones	52	2.2	54	2.7	52	2.2	55	2.4	60	2.6
C	Energy production and conversion	96	4.1	95	4.7	98	4.1	97	4.2	103	4.4
G	Carbohydrate transport and metabolism	75	3.2	76	3.7	75	3.2	75	3.2	76	3.3
E	Amino acid transport and metabolism	138	5.9	147	7.2	142	6	146	6.3	146	6.3
F	Nucleotide transport and metabolism	54	2.3	54	2.7	54	2.3	55	2.4	54	2.3
H	Coenzyme transport and metabolism	69	3	67	3.3	69	2.9	72	3.1	79	3.4
I	Lipid transport and metabolism	40	1.7	39	1.9	41	1.7	41	1.8	38	1.6
P	Inorganic ion transport and metabolism	77	3.3	76	3.7	72	3	81	3.5	87	3.7
Q	Secondary metabolites biosynthesis, transport and catabolism	17	0.7	16	0.8	16	0.7	15	0.6	17	0.7
R	General function prediction only	155	6.6	171	8.4	167	7	170	7.3	166	7.1
S	Function unknown	125	5.4	118	5.8	131	5.5	120	5.2	128	5.5
-	Not in COGs	927	40	668	33	931	39	891	39	864	37

^a^% - Percent of annotated genes. The total is based on the total number of protein coding genes in the genome

## Insights from the genome sequences

### Metabolic network analysis

The metabolic Pathway/Genome Databases (PGDBs) for strains ACC19a, CM2, and CM5 were generated on February 10, 2013 from genomic data obtained from RefSeq [17–19] by the PathoLogic program using Pathway Tools software version 17.0 [24] and MetaCyc version 17.0 [25]. These PGDBs are categorized as Tier 3, meaning that they were generated computationally, have undergone no subsequent manual curation, and may contain errors [26]. In addition, the RAST annotations of the genomic data for all five strains were uploaded to a downloadable version of Pathway Tools version 17.5 [24].

According to the RAST annotations, for strains ACC19a, CM2, and CM5, complete “sucrose degradation III (sucrose invertase)” pathways were predicted in Pathway Tools, but were marked as not present based on the RefSeq data. Based on the RAST annotations, for strains OBRC8 and AS15, this pathway was also predicted in Pathway Tools. Based on biological testing, strains CM2, OBRC8, and AS15, but not ACC19a and CM5, used sucrose as a carbon source. Strains CM2, OBRC8, and AS15 were also able to use glucose and maltose as carbon sources (Table 1). In Pathway Tools, glucose is part of multiple pathways, including glycolysis I and III, glucose and xylose degradation, and heterolactic fermentation pathways. For all five strains, there was a complete glycolysis III pathway. In Pathway Tools, maltose is also part of multiple pathways, including, the starch degradation I through V and the glycogen degradation I pathways. In the starch degradation V pathway, a 4-α-glucanotransferase (EC 2.4.1.25) is required to degrade maltose into α-D-glucose. We confirmed that strains CM2, OBRC8, and AS15 have a gene for this protein.

### Phenotypic and phylogenetic comparison

Based on 16S rRNA gene sequence comparisons, strains ACC19a, CM2, CM5, and OBRC8 are closely related to each other, with 98.9 – 99.9 % sequence identity. These four novel isolates are only distantly related to [*Eubacterium*] *yurii* subs. *yurii* and [*E.*] *yurii* subs. *schtitka,* with 93.2 – 94.4 % 16S rRNA gene sequence identity, and to *Filifactor alocis**,* with 85.5 % sequence identity (Figure 1). Strains ACC19a, CM2, CM5, and OBRC8 are sharing only 93.6 – 94.0 % of 16S rRNA gene sequence identity with strain AS15, which is below a ‘lower cut-off window’ of 95 % for the new genus differentiation [27, 28]. Predicted DNA-DNA hybridization (DDH) values [29–31] between each of the novel strains, ACC19a, CM2, CM5, and OBRC8 and strain AS15 together with [*E.*] *yurii* subsp. *margaretiae* vary between 13.8 % - 14.3 %, clearly indicating two separate taxa (Table 6).

**Table 6:** Predicted values of DNA-DNA hybridization^a^ between strains ACC19a, CM2, CM5, OBRC8, AS15 and related members of the family *Peptostreptococcaceae*

Predicted value of DDH, %	Accession	strain ACC19^a^	stain CM2	strain CM5	strain OBRC8	strain AS15	[*Eubacterium*] *yurii* subsp. *margaretiae*
strain ACC19^a^	AFZE00000000						
strain CM2	AFZF00000000	67.6					
strain CM5	AFZG00000000	84.5	68.7				
strain OBRC8	ALNK00000000	72	78.3	68.8			
strain AS15	ALJM00000000	14.2	13.8	14.3	14.3		
[*Eubacterium*] *yurii* subsp. *margaretiae*	AEES00000000	13.9	14.1	14.1	14.2	91	
*Filifactor alocis*	CP002390	14	13.1	13.8	13.9	13.2	13.1

^a^DDH values were predicted by the Genome-to-Genome Distance calculator 2.0, formula 3 [29–31]

Predicted DDH value between four strains, ACC19a, CM2, CM5, and OBRC8 varies between 67.6 and 84.5 % (Table 6), which is above or on the brink of the threshold of 70 %, the widely accepted value of relatedness used for species demarcation [28, 32, 27]. Average nucleotide identity (ANI) value between four strains varies from 95.51 to 98.31 %, which is above 95 %, the value of relatedness recommended for species delineation [33]. Both, DDH and ANI values suggest that four strains ACC19a, CM2, CM5, and OBRC8 belong to the same species.

Strain AS15 is closely related to [*E.*] *yurii* subs. *yurii,* [*E.*] *yurii* subs. *schtitka* and [*E.*] *yurii* subsp. *margaretiae* with 98.8 - 99.3 % sequence identity. The predicted DDH value of 91.0 % between strains AS15 and [*E.*] *yurii* subsp. *margaretiae* together with 16S rRNA gene sequence identity values indicates that strains AS15, [*E.*] *yurii* subsp. *margaretiae,* [*E.*] *yurii* subs. *yurii* and [*E.*] *yurii* subs. *schtitka* represented the same species (Fig. 1, Table 6).

The number of genes identified by RAST [23] in biosynthetic pathway of strains ACC19a, CM2, CM5, OBRC8, AS15 and related organisms is shown in Table 7. Eight to nine genes associated with synthesis of teichoic and lipoteichoic acids, as annotated by RAST, were found in the genomes of strains ACC19a, CM2, CM5, and OBRC8; nine to eleven were found in the genomes of AS15 and [*E.*] *yurii* subsp. *margaretiae*; and four were found in the genome of *F. alocis* (Table 7). We detected one gene associated with synthesis of benzoquinones or naphthoquinones in genomes of strain AS15, [*E.*] *yurii* subsp. *margaretiae* only. There were no predicted gene sequences with recognizable homology to mycolic acids or lipopolysaccharides biosynthesis. Three and six RAST-annotated genes associated with diaminopimelic acid (DAP) synthesis were present in the genome of strains ACC19a, CM2, CM5, OBRC8, and AS15 and [*E.*] *yurii* subsp. *margaretiae*, respectively. According to the RAST annotations, eight to nine genes associated with polyamines metabolism, and eleven to eighteen genes, that are associated with polar lipids metabolism, were present in the genomes (Table 7).

**Table 7:** Number of genes identified in biosynthetic pathway^a^ from whole genome sequences of strains ACC19a, CM2, CM5, OBRC8, AS15 and related organisms from the family *Peptostreptococcaceae*

Genes responsible for biosynthesis	strain ACC19^a^	strain CM2	strain CM5	strain OBRC8	strain AS15	[*Eubacterium*] *yurii* subsp. *margaretiae*	*Filifactor alocis*
Accession number	AFZE00000000	AFZF00000000	AFZG00000000	ALNK00000000	ALJM00000000	AEES00000000	CP002390
Teichoic and lipoteichoic acids	9	8	8	8	9	11	4
Benzoquinones or naphthoquinones	0	0	0	0	1	1	0
Polar lipids	13	11	11	11	15	18	14
Lipopolysaccharides	0	0	0	0	0	0	0
Mycolic acids	0	0	0	0	0	0	0
Polyamines	8	8	9	8	9	9	10
Diaminopimelic acid	3	3	3	3	6	6	0

^a^Identified by Rapid Annotation Subsystem Technology (RAST)

Physiological and genomic characteristics of four novel isolates ACC19a, CM2, CM5, and OBRC8 were considerably different from the properties of strain AS15 and [*E.*] *yurii* subs. *yurii*, [*E.*] *yurii* subs. *schtitka*, and [*E.*] *yurii* subsp. *margaretiae* [13, 14]. Strains ACC19a, CM2, CM5, OBRC8 were represented by highly motile peritrichous rods with round ends, single or in short chains; while strain AS15, [*E.*] *yurii* subs. *yurii*, [*E.*] *yurii* subs. *schtitka*, and [*E.*] *yurii* subsp. *margaretiae* were straight rods with single subpolar flagellum and square ends, that formed rosettes or brush-like aggregates. Contrary to strain AS15, [*E.*] *yurii* subs. *yurii*, [*E.*] *yurii* subs. *schtitka* and [*E.*] *yurii* subsp. *margaretiae*, strains ACC19a, CM2, CM5, and OBRC8 did not produce indole. In addition, strain AS15 showed alpha-hemolytic activity on blood TY-agar medium, while strains ACC19a, CM2, CM5, and OBRC8 were non-hemolytic. Metabolic end products of glucose fermentation of [*E.*] *yurii* subs. *yurii* and [*E.*] *yurii* subs. *schtitka* and [*E.*] *yurii* subsp. *margaretiae* were butyrate, acetate and propionate; strains ACC19a, CM2, CM5, and OBRC8 produced acetate and propionate only.

DNA G + C content of strains ACC19a, CM2, CM5, and OBRC8 was 30 – 30.68 %, while G + C of strain AS15, [*E.*] *yurii* subs. *yurii* and [*E.*] *yurii* subs. *schtitka* and [*E.*] *yurii* subsp. *margaretiae* was 32 -32.24 %.

## Conclusions

Unique phenotypic, phylogenetic, and genomic features allow for the differentiation of strains ACC19a, CM2, CM5, and OBRC8 from strain AS15, [*E.*] *yurii* subs. *yurii*, [*E.*] *yurii* subs. *schtitka*, [*E.*] *yurii* subsp. *margaretiae* and *F. alocis*. Based on the distinct characteristics presented, we suggest that strains ACC19a, CM2, CM5, OBRC8 represent a novel genus and species within the family *Peptostreptococcaceae*, for which we propose the name *Peptoanaerobacter stomatis* gen. nov., sp. nov. The type strain is strain ACC19a^T^ (=HM-483^T^; =DSM 28705^T^; =ATCC BAA-2665^T^).

### Description of Peptoanaerobacter gen. nov.

*Peptoanaerobacter* (Gr. v. *peptô*, cook, digest; Gr. pref. *an*-, not; Gr. masc. n. *aer*, air; N.L. masc. n. *bacter*, rod, staff; N.L. masc. n. *anaerobacter*, the digesting rod not [living] in air).

Cells are Gram-positive, structurally and after staining, motile peritrichous rods with round ends, about 1.2 – 2.5 μm long and 0.4 – 0.8 μm wide, often occurring in chains. No spores are formed. Strictly anaerobic. Catalase, oxidase and urease are negative. Nitrate is not reduced. Growth is supported by yeast extract but not Casamino acids. Yeast extract is required for growth on glucose, sucrose and maltose. The major metabolic end-products of glucose fermentation are acetate and propionate. Growth temperature range is 30–42 ^o^C. Major fatty acids are C14:0, C16:0, C16:1ω 7c. Genes responsible for biosynthesis of teichoic and lipoteichoic acids, polar lipids, polyamines and DAP are present in the genome. There are no genes responsible for biosynthesis of respiratory benzoquinones or naphthoquinones, mycolic acids or lipopolysaccharides. The type species is *Peptoanaerobacter stomatis*.

### Description of Peptoanaerobacter stomatis sp. nov. Gr. n. stoma stomatos, mouth; N.L. gen. n. stomatis, of the mouth

Cell morphology is as described for the genus. Colonies are pin-point, circular, convex beige, 0.5 mm in diameter, and non-hemolytic. Acid is produced from glucose, maltose and sucrose, but not lactose, arabinose, cellobiose, mannose, melezitose, raffinose, rhamnose, trehalose, xylose, glycerol, mannitol, salicin and sorbitol. Indole is not produced. Gelatin is not liquefied. Esculin is not hydrolyzed. The type strain is susceptible to discs containing 1 mg kanamycin, 2 units penicillin, 60 μg erythromycin, 30 μg chloramphenicol, 30 μg tetracycline and bile. The genome is 2,541,543-bp long and contains 2,277 protein-coding and 54 RNA genes. DNA G + C content is 30.37 mol %. The type strain ACC19a (=DSM 28705^T^; =HM-483^T^; =ATCC BAA-2665^T^) was isolated from the human subgingival dental plaque. Habitat: human mouth.

## Additional file

Additional file 1: Table S1. Associated MIGS records of *Peptostreptococcaceae* spp. ACC19a, CM2, CM5, OBRC8, and AS15.

## Abbreviations

HMP: Human Microbiome Project
HOMD: Human Oral Microbiome Database
DDH: DNA-DNA hybridization
BEI Resources: Biodefense and Emerging Infections Research Resources Repository
DSMZ: Deutsche Sammlung von Mikroorganismen und Zellkulturen GmbH (German Collection of Microorganisms and Cell Cultures GmbH)
GOLD: Genome On-Line Database
IMG: Integrated Microbial Genomes
RAST: Rapid Annotation using Subsystem Technology
