# Supplementary material for: High-quality draft genome sequences of five anaerobic oral bacteria and description of Peptoanaerobacter stomatis gen. nov., sp. nov., a new member of the family Peptostreptococcaceae
Source: Stand Genomic Sci. 2015 Jul 18;10:37. doi: 10.1186/s40793-015-0027-8 (PMC4517659; doi:10.1186/s40793-015-0027-8)
Supplement: Additional file 1: Table S1. — Associated MIGS records of Peptostreptococcaceae spp. ACC19a, CM2, CM5, OBRC8, and AS15. [file 40793_2015_27_MOESM1_ESM.pdf]

Table S1. Associated MIGS records of *Peptostreptococcaceae* spp. ACC19a, CM2, CM5, OBRC8, and AS15.

MIGS ID	field name	description				
		strain ACC19a	strain CM2	strain CM5	strain OBRC8	strain AS15
MIGS-1	Submit to INSDC/Trace archives					
1.1	PID	49887	49889	49891	78565	78563
1.2	GCAT_ID	007931_GCAT	007932_GCAT	007930_GCAT	010963_GCAT	010962_GCAT
	NCBI Taxon ID	796937	796939	796940	796941	1115804
	Genome Data	AFZE00000000	AFZF00000000	AFZG00000000	ALNK00000000	ALJM00000000
	HMP_ID	9629	9630	9628	1143	1142
	HOMD ID	HOMD: tax_081	HOMD: tax_081	HOMD: tax_081	HOMD: tax_081	HOMD: tax_377
	IMG Object ID	2513237269	2562617100	2513237271	2531839269	2537561888
MIGS-2	MIGS CHECK LIST TYPE					
	Domain	Bacteria	Bacteria	Bacteria	Bacteria	Bacteria
	Phylum	Firmicutes	Firmicutes	Firmicutes	Firmicutes	Firmicutes
	Class	Clostridia	Clostridia	Clostridia	Clostridia	Clostridia
	Order	Clostridiales	Clostridiales	Clostridiales	Clostridiales	Clostridiales
	Family	Peptostreptococcaceae	Peptostreptococcaceae	Peptostreptococcaceae	Peptostreptococcaceae	Peptostreptococcaceae
MIGS-3	Project Name	Peptostreptococcaceae sp. ACC19a	Peptostreptococcaceae sp. CM2	Peptostreptococcaceae sp. CM5	Peptostreptococcaceae sp. OBRC8	Peptostreptococcaceae sp. AS15
	Genus	unclassified	unclassified	unclassified	unclassified	[*Eubacterium*]
	Species	unclassified	unclassified	unclassified	unclassified	[*Eubacterium*] *yurii*
	Subspecies	unclassified	unclassified	unclassified	unclassified	*margaretiae*
	Strain	ACC19a	CM2	CM5	OBRC8	AS15
	NCBI status	complete	complete	complete	complete	complete
MIGS-4	Geographic Location	Boston, MA, USA	Boston, MA, USA	Boston, MA, USA	Boston, MA, USA	Boston, MA, USA
4.1	Latitude	42° 21′ N	42° 21′ N	42° 21′ N	42° 21′ N	42° 21′ N
4.2	Longitude	71° 03′ W	71° 03′ W	71° 03′ W	71° 03′ W	71° 03′ W
4.4	Altitude	43 m	43 m	43 m	43 m	43 m
4.5	Isolation Site	Human oral cavity	Human oral cavity	Human oral cavity	Human oral cavity	Human oral cavity
4.6	Isolation	USA	USA	USA	USA	USA

	Country					
	Isolation Source	Maria Sizova and Slava Epstein	Maria Sizova and Slava Epstein	Maria Sizova and Slava Epstein	Maria Sizova and Slava Epstein	Maria Sizova and Slava Epstein
MIGS-5	Sample Collection Date	March 2010	March 2010	March 2010	March 2010	March 2010
MIGS-6	Habitat	Human oral cavity	Human oral cavity	Human oral cavity	Human oral cavity	Human oral cavity
6.1	temperature	37˚ C	37˚ C	37˚ C	37˚ C	37˚ C
6.2	pH	Neutral	Neutral	Neutral	Neutral	Neutral
6.3	salinity	Normal	Normal	Normal	Normal	Normal
MIGS-7	Subspecific genetic lineage	not reported	not reported	not reported	not reported	not reported
MIGS-9	Number of replicons	1	1	1	1	1
MIGS-10	Plasmid Count	0	0	0	0	0
MIGS-11	Estimated Size	2542 kb	2313 kb	2594 kb	2553 kb	2655 kb
	ORFS	2331	2030	2379	2313	2336
	Chromosome Count	1	1	1	1	1
MIGS-12	Isolation Pubmed ID	PMID: 22057871	PMID: 22057871	PMID: 22057871	PMID: 22057871	PMID: 22057871
MIGS-13	Culture Collection	BEI HM-483	BEI HM-484	BEI HM-485	BEI HM-765	BEI HM-766
	Goldstamp	Gi06852	Gi06853	Gi06851	Gi09663	Gi09662
	Publication	PMID: 22057871	PMID: 22057871	PMID: 22057871	PMID: 22057871	PMID: 22057871
MIGS-14	Pathogenicity	not reported	not reported	not reported	not reported	not reported
MIGS-15	Biotic Relationship	Commensal	Commensal	Commensal	Commensal	Commensal
MIGS 16	Host Name	Homo sapiens	Homo sapiens	Homo sapiens	Homo sapiens	Homo sapiens
	Host Taxon ID	9606	9606	9606	9606	9606
	Host Gender	Female	Female	Female	Female	Female
	Host Age	25	24	24	21	27
	Host Race	African-American	Caucasian	Caucasian	Caucasian	African-American
MIGS-17	Host specificity or range (taxid)	Oral cavity	Oral cavity	Oral cavity	Oral cavity	Oral cavity
	Body Sample Site	Oral cavity	Oral cavity	Oral cavity	Oral cavity	Oral cavity

	Body Product	Dental plaque	Dental plaque	Dental plaque	Dental plaque	Dental plaque
	Body Sample Subsite	Subgingival	Subgingival	Subgingival	Subgingival	Subgingival
	Host Comments	not reported	not reported	not reported	not reported	not reported
MIGS-18	Host Health	Healthy	Healthy	Healthy	Healthy	Healthy
MIGS-19	Trophic Level	Commensal	Commensal	Commensal	Commensal	Commensal
MIGS-22	Relationship to Oxygen	Strictly anaerobic	Strictly anaerobic	Strictly anaerobic	Strictly anaerobic	Strictly anaerobic
	Carbon Source	Yeast extract	Yeast extract	Yeast extract, Glucose, Sucrose, Maltose	Yeast extract, Glucose, Sucrose, Maltose	Yeast extract, Glucose, Sucrose, Maltose
MIGS-23	Isolation and Growth conditions	PMID: 22057871	PMID: 22057871	PMID: 22057871	PMID: 22057871	PMID: 22057871
MIGS 28	Library construction	Two 454 pyrosequencing libraries: one standard 0.6kb fragment library and one 2.5kb jump library	Two 454 pryosequencing libraries: one standard 0.6kb fragment library and one 2.5kb jump library. Two Illumina libraries: one standard 180bp fragment library and one 3-5kb jump library	Two 454 pyrosequencing libraries: one standard 0.6kb fragment library and one 2.5kb jump library	Standard Paired End Illumina library generated for HiSEQ2000	Standard Paired End Illumina library generated for HiSEQ2000
28.1	Library Method	0.6kb and 2.5kb	0.6kb and 2.5kb 180bp and 3-5kb	0.6kb and 2.5kb	0.45 kb paired end library	0.45 kb paired end library
28.2	Reads Count	337046	25246427	354415	7983051	865772
MIGS-29	Sequencing platforms	454	454; Illumina HiSeq 2000	454	Illumina HiSeq 2000	Illumina HiSeq 2000
MIGS-30	Assemblers	Newbler v. 2.3 PostRelease-11/19/2009	Newbler v. 2.3 PostRelease-11/19/2009 and ALL-PATHS version R39099	Newbler v. 2.3 PostRelease-	Newbler v. 2.3 PostRelease-11/19/2009	Newbler v. 2.3 PostRelease-11/19/2009 and ALL-PATHS version R39099
30.2	estimated error rate	less than 1 in 10,000 bp	less than 1 in 1,000,000 bp	less than 1 in 10,000 bp	Less than 3%	Less than 3%

30.3	method of calculation				utgErrorRate calculated value with Celera Assembler	utgErrorRate calculated value with Celera Assembler
MIGS-31	Finishing Quality	High Quality Draft	High Quality Draft	High Quality Draft	High Quality Draft	High Quality Draft
31.1	Sequencing Status	Complete	Complete	Complete	Complete	Complete
	Sequencing Quality	Level 2: High-Quality Draft	Level 2: High-Quality Draft	Level 2: High-Quality Draft	Level 2: High-Quality Draft	Level 2: High-Quality Draft
31.2	Fold Coverage	40×	282×	39×	32×	31×
31.3	Contig Count	59	19	106	40	52
MIGS-32	Relevant SOPs	not reported	not reported	not reported	not reported	not reported
MIGS-33	Genome Database	Human Microbiome Project (HMP)	Human Microbiome Project (HMP)	Human Microbiome Project (HMP)	Human Microbiome Project (HMP)	Human Microbiome Project (HMP)
MIGS-34	Gene calling Method	PRODIGAL	PRODIGAL	PRODIGAL	GLIMMER	GLIMMER
MIGS-35	GC Content	30%	30%	30%	31%	32%
MIGS-36	Project Status	Complete	Complete	Complete	Complete	Complete
	Availability	Public	Public	Public	Public	Public
	Sequencing Centers	Broad Institute	Broad Institute	Broad Institute	JCVI	JCVI
	Funding	NIH	NIH	NIH	NIH	NIH
	Contact Name	Broad Institute	Broad Institute	Broad Institute	JCVI	JCVI
MIGS-37						
37.1	Cell Shape	Rods with round ends	Rods with round ends	Rods with round ends	Rods with round ends	Rods with square ends
37.2	Motility	Motile	Motile	Motile	Motile	Motile
37.3	Sporuation	Non-sporulating	Non-sporulating	Non-sporulating	Non-sporulating	Non-sporulating
37.4	Pressure	atmospheric	atmospheric	atmospheric	atmospheric	atmospheric
37.5	Cell Diameter (um)	0.4 - 0.8	0.5 - 0.7	0.5 - 0.7	0.6 - 0.8	0.4 - 0.5
37.6	Cell Length (um)	1.2 - 2.5	1.0 - 2.3	1.3 - 2.7	1.4 - 3.5	1.5 - 4.7
37.7	Color	not reported	not reported	not reported	not reported	not reported

37.8	Gram Staining	Gram-positive	Gram-positive	Gram-positive	Gram-positive	Gram-positive
37.9	Cell arrangement	individual cells, some in chains	individual cells, some in chains	individual cells, some in chains	individual cells, some in chains	individual cells, some in rosettes
	Disease	Unknown	Unknown	Unknown	Unknown	Unknown
37.10	Metabolism	acetate and propionate producer	acetate and propionate producer	acetate and propionate producer	not reported	not reported
37.12	Temperature Range	Mesophile	Mesophile	Mesophile	Mesophile	Mesophile
MIGS-38						
38.1	Contact Name	Ashlee Earl	Ashlee Earl	Ashlee Earl	Manolito Torralba	Manolito Torralba
38.2	Contact email	hmpstrainsbroad@ broad.mit.edu	hmpstrainsbroad@ broad.mit.edu	hmpstrainsbroad@ broad.mit.edu	hmpstrains@jcvi.org	hmpstrains@jcvi.org
	Locus Tag	HMPREF9629	HMPREF9630	HMPREF9628	HMPREF1143	HMPREF1142
	Completion Date	2014-01-09	2014-01-09	2014-01-09	2012-08-27	2014-01-05
	Publication	PMID: 22057871	PMID: 22057871	PMID: 22057871	PMID: 22057871	PMID: 22057871
	Project Relevance	Human Microbiome Project (HMP)	Human Microbiome Project (HMP)	Human Microbiome Project (HMP)	Human Microbiome Project (HMP)	Human Microbiome Project (HMP)
